# Structural Basis of Beneficial Design for Effective Nicotinamide Phosphoribosyltransferase Inhibitors

**DOI:** 10.3390/molecules25163633

**Published:** 2020-08-10

**Authors:** Sei-ichi Tanuma, Kiyotaka Katsuragi, Takahiro Oyama, Atsushi Yoshimori, Yuri Shibasaki, Yasunobu Asawa, Hiroaki Yamazaki, Kosho Makino, Miwa Okazawa, Yoko Ogino, Yoshimi Sakamoto, Miyuki Nomura, Akira Sato, Hideaki Abe, Hiroyuki Nakamura, Hideyo Takahashi, Nobuhiro Tanuma, Fumiaki Uchiumi

**Affiliations:** 1 Department of Genomic Medicinal Science, Research Institute for Science and Technology, Organization for Research Advancement, Tokyo University of Science, Noda, Chiba 278-8510, Japan; miwa.okazawa@rs.tus.ac.jp; 2 Department of Biochemistry, Faculty of Pharmaceutical Sciences, Tokyo University of Science, Noda, Chiba 278-8510, Japan; 3b12023@alumni.tus.ac.jp (K.K.); yryr.sbsk040809@gmail.com (Y.S.); ogino@rs.tus.ac.jp (Y.O.); akirasat@rs.tus.ac.jp (A.S.); 3 Hinoki Shinyaku Co., Ltd., Chiyoda-ku, Tokyo 102-0084, Japan; takahiro.oyama@hinoki.co.jp (T.O.); hymanami@gmail.com (H.Y.); hideaki.abe@hinoki.co.jp (H.A.); 4 Institute for Theoretical Medicine Inc., Fujisawa, Kanagawa 251-0012, Japan; yoshimori@itmol.com; 5 Laboratory for Chemistry and Life Science, Institute of Innovative Research, Tokyo Institute of Technology, Yokohama, Kanagawa 226-8503, Japan; asawa.y.aa@m.titech.ac.jp (Y.A.); hiro@res.titech.ac.jp (H.N.); 6 Department of Medicinal Chemistry, Faculty of Pharmaceutical Sciences, Tokyo University of Science, Noda, Chiba 278-8510, Japan; kosho-maki@rs.tus.ac.jp (K.M.); hide-tak@rs.tus.ac.jp (H.T.); 7 Department of Gene Regulation, Faculty of Pharmaceutical Sciences, Tokyo University of Science, Noda, Chiba 278-8510, Japan; f_uchiumi@rs.tus.ac.jp; 8 Division of Cancer Chemotherapy, Miyagi Cancer Center Research Institute, Natori, Miyagi 981-1293, Japan; yoshimi.sk.88@gmail.com (Y.S.); nomu.miyu@gmail.com (M.N.); ntanuma@med.tohoku.ac.jp (N.T.)

**Keywords:** nicotinamide phosphoribosyltransferase, NAD^+^ biosynthesis, inhibitor, azacyclohexane, anticancer drug, drug design, enthalpy effect

## Abstract

Inhibition of nicotinamide phosphoribosyltransferase (NAMPT) is an attractive therapeutic strategy for targeting cancer metabolism. So far, many potent NAMPT inhibitors have been developed and shown to bind to two unique tunnel-shaped cavities existing adjacent to each active site of a NAMPT homodimer. However, cytotoxicities and resistances to NAMPT inhibitors have become apparent. Therefore, there remains an urgent need to develop effective and safe NAMPT inhibitors. Thus, we designed and synthesized two close structural analogues of NAMPT inhibitors, azaindole–piperidine (**3a**)- and azaindole–piperazine (**3b**)-motif compounds, which were modified from the well-known NAMPT inhibitor FK866 (**1**). Notably, **3a** displayed considerably stronger enzyme inhibitory activity and cellular potency than did **3b** and **1**. The main reason for this phenomenon was revealed to be due to apparent electronic repulsion between the replaced nitrogen atom (N1) of piperazine in **3b** and the Nδ atom of His191 in NAMPT by our in silico binding mode analyses. Indeed, **3b** had a lower binding affinity score than did **3a** and **1**, although these inhibitors took similar stable chair conformations in the tunnel region. Taken together, these observations indicate that the electrostatic enthalpy potential rather than entropy effects inside the tunnel cavity has a significant impact on the different binding affinity of **3a** from that of **3b** in the disparate enzymatic and cellular potencies. Thus, it is better to avoid or minimize interactions with His191 in designing further effective NAMPT inhibitors.

## 1. Introduction

Nicotinamide adenine dinucleotide (NAD^+^) is a critical molecule in control of numerous basic cellular processes, such as ATP production and maintenance of cellular integrity and genome stability [1,2,3,4]. It not only serves as a cofactor for redox bioreactions but can also be a substrate for multiple NAD^+^-consuming enzymes, such as poly(ADP-ribose) polymerases, mono-ADP-ribosyltransferases and sirtuins, participating in the epigenetic regulation of DNA transaction (transcription, replication, repair and recombination), cellular signaling processes and calcium homeostasis, and thereby in cell proliferation, differentiation and death [3,4,5,6,7,8]. Given the biological importance of this molecule, mammalian cells have evolved multiple biosynthetic pathways to produce NAD^+^; it is synthesized from tryptophan, nicotinic acid and nicotinamide (NAM)/nicotinamide riboside via *de novo*, Preiss–Handler and salvage pathways, respectively [1,2,3,4,9,10,11].

As NAD^+^ is consumed through many enzymatic processes, accelerated NAD^+^ depletion is often characteristic in cancer cells [9,10,11,12]. To rapidly replenish the NAD^+^ pool, cancer cells rely heavily on the NAM salvage pathway that backs NAM to NAD^+^ in two steps primarily catalyzed by nicotinamide phosphoribosyltransferase (NAMPT) [13,14,15,16,17]. Furthermore, in various types of cancer cells, NAMPT is found to be up-regulated [8,13,16,17], although the molecular mechanisms that dictate the salvage pathway choice remain elusive. Thus, NAMPT is considered an attractive target for the development of new anticancer drugs and therapies [18,19,20,21,22,23,24,25,26,27,28,29,30,31].

**Figure 1:**
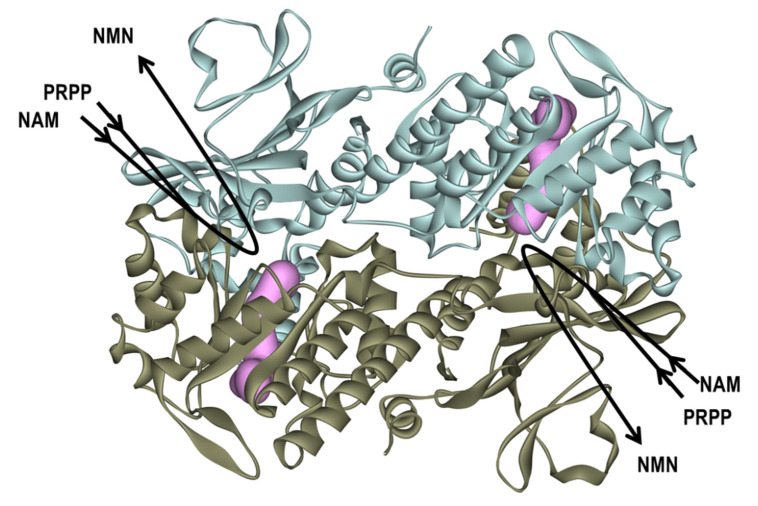
Overview of the human (h) nicotinamide phosphoribosyltransferase (NAMPT) as the dimer form and its enzyme reaction. The homodimer structure of hNAMPT (PDB Code: 2GVJ) [38,39,40,41,42] is shown in the ribbon diagram colored light blue and brown, respectively. The two tunnel-shaped cavities near the active sites in the hNAMPT molecule are shown in magenta shading. NAMPT catalyzes the conversion of nicotinamide (NAM) and phosphoribosyl pyrophosphate (PRPP) to produce nicotinamide mononucleotide (NMN).

NAMPT, which functions as a homodimer with two unique tunnel-shaped cavities existing adjacent to each active site at the dimer interface, catalyzes the rate-limiting primary step of the salvage pathway, the transfer of phosphoribosyl residue from 5-phosphoribosyl-1-pyrophosphate (PRPP) to NAM to produce nicotinamide mononucleotide (NMN) (Figure 1) [1,2,3,4,32,33,34]. Many NAMPT inhibitors, such as FK866 (**1**), CSH-828, GNE0617 and STF-11880, which bind to the tunnel cavity of NAMPT, have been reported to exert anti-tumor suppression effects and have progressed to clinical trials [18,23,24,25,30]. However, cytotoxicities dominated by gastrointestinal symptoms and thrombocytopenia and resistance to NAMPT inhibitors have become apparent [23,35,36,37]. Therefore, there remains an urgent need to develop effective and safe anticancer NAMPT inhibitors for cancer chemotherapy. Thus, detailed insights into the tunnel structure of NAMPT are required to generate effective NAMPT inhibitors with a better therapeutic index and that can overcome resistance. 

In the present study, we designed and synthesized two close structural analogues of NAMPT inhibitors **3a** and **3b**, which were modified from the best explored NAMPT inhibitor 1 [18,35]. Interestingly, we showed that **3a** has a considerably stronger enzyme inhibitory activity and cellular potency than does **3b** or **1**. Furthermore, using these inhibitors as chemical probes, we characterized the inhibitor-targeting tunnel cavity of NAMPT. Importantly, our in silico binding mode analyses of these inhibitors with the tunnel cavity of NAMPT revealed that there is apparent electronic repulsion only between the nitrogen atom of piperazine in **3b** and the Nδ atom of His191 in NAMPT. These findings indicate that the electrostatic enthalpy potential inside the tunnel region has a significant impact on the different binding affinity of **3a** from **3b** in the disparate cellular potencies. Thus, these results provide new insights into the design for further effective NAMPT inhibitors for cancer chemotherapies.

## 2. Results and Discussion

### 2.1. Synthesis and Biochemical Properties of **3a** and **3b**

**Figure 2:**
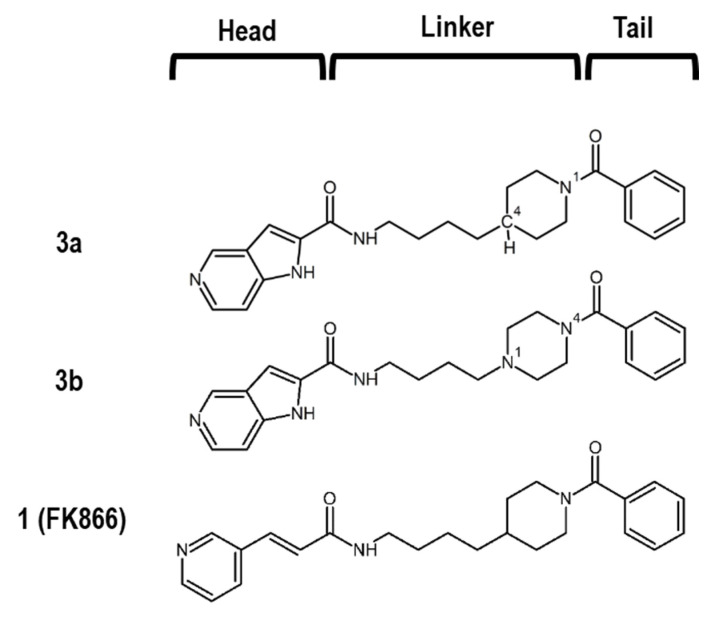
Chemical structures of **3a**, **3b** and **1**.

**Scheme 1:**
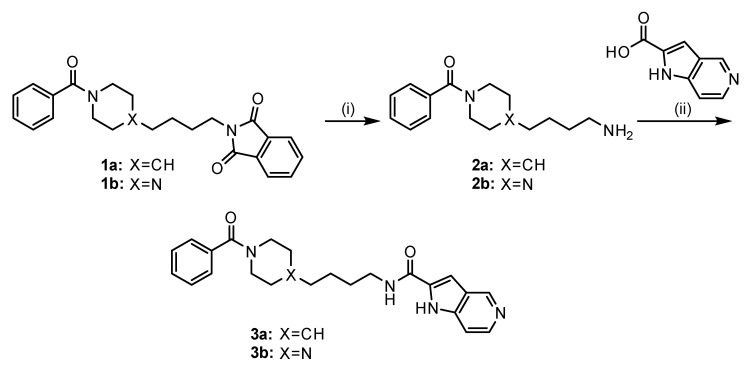
Synthetic route of compounds **3a** and **3b**. Reagents and conditions: (**i**) NH_2_NH_2_, EtOH, r.t. to reflux, 2 h; (**ii**) 1*H*-pyrro[3,2-c]pyridine-2-carboxylic acid, EDC-HCl, DIPEA, DMF, r.t., 24 h. The targets **3a** and **3b** were prepared according to the methods of [45,46], respectively, with slight modifications.

Many NAMPT inhibitors bind to the tunnel-shaped cavity of NAMPT (Figure 1), although the role of this tunnel in NAMPT function remains unknown [18,19,20,21,22,23,24,25,26,27,28,29,30,31]. These inhibitors have a unique pharmacophore consisting of three parts (Figure 2): head (a pyridine-like aromatic moiety), linker (a tunnel-interacting moiety) and tail (a solvent-exposed bulky group). To get more valuable insights into the tunnel cavity for the development of effective NAMPT inhibitors, we structure-basically designed and synthesized two close structural analogues of NAMPT inhibitors, namely azaindole–piperidine (**3a**)- and azaindole–piperazine (**3b**)-motif compounds, which were modified from the head and linker moieties of **1** (Figure 2, Scheme 1). Since the tail acts as a packing moiety against the solvent exposed tunnel exit surface, the tail structures of **3a** and **3b** were fixed as the same benzoyl group of **1**. The nitrogen-containing aromatic heads present in many NAMPT inhibitors mimic the natural NAM substrate of the enzyme [18,19,20,21,22,23,24,25,26,27,28,29,30,31]. However, the head moiety of vinylpyridine ring of **1** (Figure 2) is known to be able to non-specifically interact with proteins [43,44]. Based on this knowledge, we first replaced this head to the 5-azaindole heterocyclic ring to improve the specificity to NAMPT (Figure 2). Secondly, as the linker moiety of azacyclohexane may be considered to become a critical motif for NAMPT inhibitory activity, we synthesized a close structural set of piperidine (**3a**)- and piperazine (**3b**)-motif NAMPT inhibitors according to the methods of Bair et al. [45] and Vogel et al. [46], respectively (Scheme 1).

**Figure 3:**
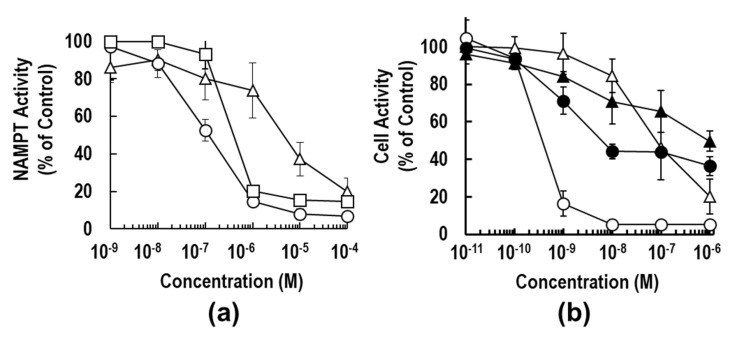
Biochemical properties of **3a** and **3b**. (**a**) The inhibitory activities of **3a** (open circle), **3b** (open triangle) and **1** (open square) against NAMPT enzyme were examined, as described previously [31,36,37]. NAMPT activity was measured using a coupled-enzyme reaction system (CycLex NAMPT colorimetric assay kit) in 96-well plate format using the one-step method. Data are the averages of three independent experiments, and the bars indicate the standard error (SE) values. (**b**) The anti-proliferative effects of **3a** (circle) and **3b** (triangle) on HCT116 cells were investigated as described previously [31,36,37]. HCT116 cells were treated with the indicated concentrations of each inhibitor with (closed symbol) or without (open symbol) 100 μM NMN for 72 h. The cell activity (% of control) was measured by use of the WST-8 assay. Data are presented as the means of three independent experiments ± SE, and the bars indicate the SE values.

**Figure 4:**
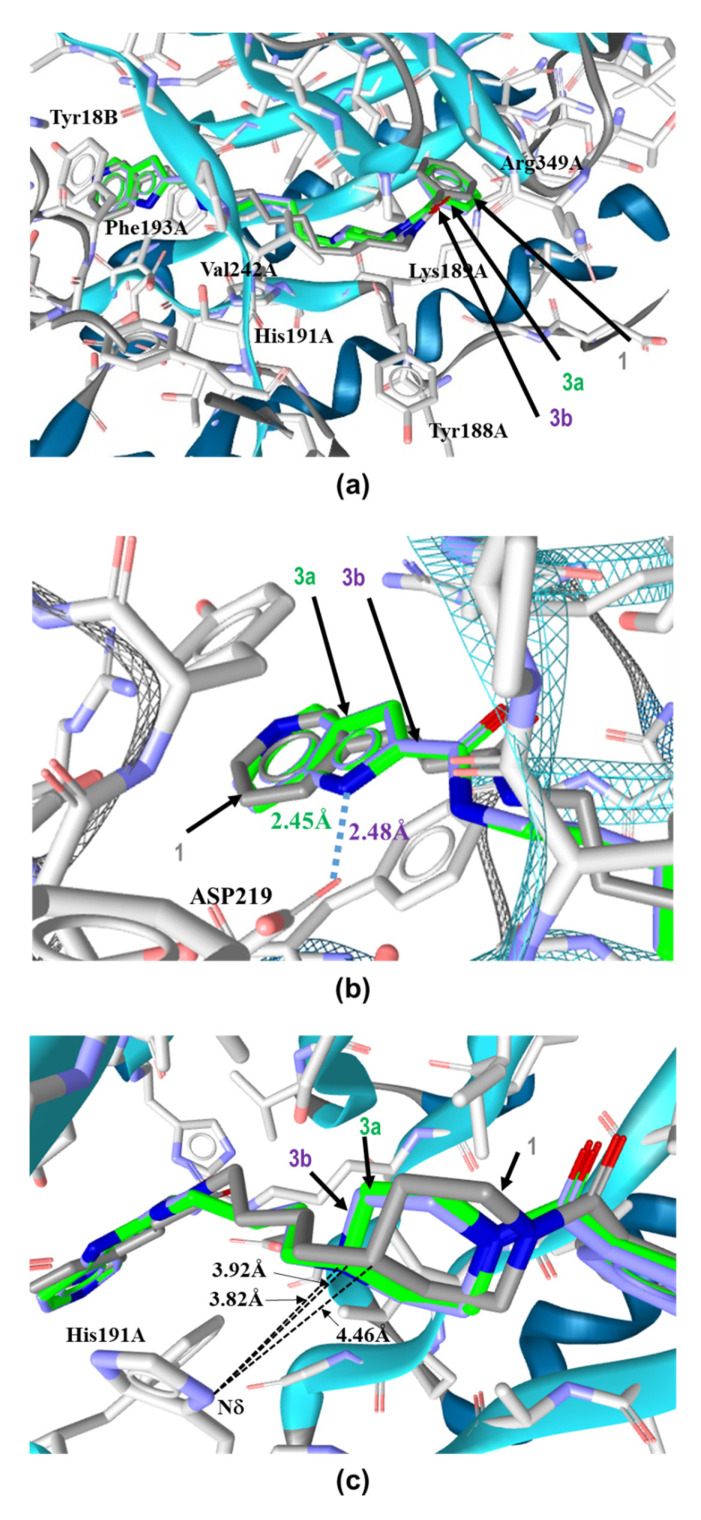
Binding modes of **3a**, **3b** and **1** in the tunnel cavity of NAMPT. (**a**) Binding modes of **3a, 3b** and **1** in the tunnel cavity of NAMPT were analyzed by our in silico binding mode analysis using RDKit [48] and LigandScout [49]. (**b**) Close-up view of head regions of these three NAMPT inhibitors in the tunnel cavity of NAMPT using Open3DALIGN [53]. (**c**) Close-up view of the binding interactions of azacyclohexane-linker moieties of these NAMPT inhibitors with His191A located in the tunnel cavity of NAMPT. Carbons are colored green (**3a**), magenta (**3b**) and gray (**1**). Nitrogen and oxygen are colored blue and red, respectively.

To compare the NAMPT inhibitory activities of **3a**, **3b** and **1**, we determined the IC_50_ values by the titration curves from the standard calorimetric enzyme-cycling assay [31,36,37]. As shown in Figure 3a, the inhibitory activity of **3a** (IC_50_ = 0.11 μM) was approximately 5-fold stronger than that of **1** (IC_50_ = 0.52 μM). This result suggests that the replacement of vinylpyridine with an azaindole ring could improve anti-NAMPT activity. The binding mode analyses of these inhibitors on the NAMPT molecule support this observation, as described in the next section; a hydrogen bond formed between the N1 atom of azaindole and Asp219 of NAMPT (Figure 4b). Surprisingly, the substitution of piperidine (**3a**) with piperazine (**3b**) resulted in a dramatic loss in inhibitory activity against NAMPT; the inhibitory activity of **3b** (IC_50_ = 5.06 μM) was shown to be about 50-fold weaker than that of **3a**. The inhibition curve of **3a** was much steeper than that of **3b**, suggesting that incubation with **3a** resulted in the formation of an entity capable of extremely tight binding to the tunnel cavity of NAMPT. From these observations, we suspect that unfavorable interactions between the piperazine moiety of **3b** and the tunnel cavity of NAMPT were responsible for the considerably weak anti-NAMPT activity.

To confirm the marked differences of anti-NAMPT activities between **3a** and **3b**, the anti-proliferation effects of these inhibitors on the human colon cancer cell line HCT116 were examined after continuous exposure for 72 h [31,36,37]. As shown in Figure 3b, compound **3b** displayed significantly weaker cell activity; the 50% effective concentration (EC_50_) value was calculated to be 87.5 nM, which was approximately 200-fold higher than that of **3a** (EC_50_ = 0.48 nM). These results of the different inhibition degrees in cell-based assay from those in enzyme assay (Figure 3) paralleled observations by others that the degrees of potency of cell-based assessments were not always identical with those of NAMPT enzymatic inhibition [26,28,47]. In addition, there was little cytotoxicity on non-cancer cell lines, such as TIG-120, WI38 and MRC-5 cells. Both **3a** and **3b** caused more than 90% reduction of NAD^+^ levels in the cancer cells after 24 h-treatment and thereby induced cell death. Furthermore, supplementation of the product of NAMPT, NMN, to the cell culture medium could significantly rescue HCT116 cells from cell death by treatment with **3a** or **3b** (Figure 3b). Additionally, the dose–response curves of **1** were similar to those of **3a**. These results confirmed that NAMPT is a target of both inhibitors. Collectively, these results indicate that the substitution of one carbon (C) atom of the piperidine moiety in **3a** for a nitrogen (N) atom (piperazine moiety in **3b**) exerts a critical effect on the anti-NAMPT activity.

### 2.2. Binding Modes of **3a** and **3b** to the Tunnel Cavity of NAMPT

To understand the different NAMPT inhibitory activities of **3a** and **3b**, we analyzed their binding modes to NAMPT (PDB Code: 2GVJ [38,39,40,41,42]) by our in silico binding mode analyses using RDKit [48] and LigandScout [49]. The superimpositions of these inhibitors in the tunnel cavity revealed that they adopted almost identical chair binding forms as with the case in **1** (Figure 4a). The NAMPT residues that contacted these inhibitors were observed in very similar locations in the structure. These results indicate that the binding modes of **3a** and **3b** in the tunnel cavity of NAMPT do not significantly alter as compared with that of **1**.

When observed closely, the position of the head regions of **3a** and **3b** in the tunnel cavity were nearly identical to that of **1** (Figure 4b). The subtle shifts observed in **3a** and **3b** from the position of **1** may have been introduced by their slightly different orientations of the head-azaindole bicyclic ring from the head-pyridine ring of **1** inside the tunnel cavity of NAMPT. The azaindole rings of **3a** and **3b** were sandwiched between Phe193A and Tyr18B, forming tight aromatic π–π stacks like the pyridine ring of **1**. Additionally, the nitrogen atoms (N1) in the azaindole rings of **3a** and **3b** could interact with Asp219 of NAMPT by a hydrogen bond (Figure 4b), thus suggesting that they might be involved in the tighter specific binding of **3a** and **3b** to NAMPT than that of **1**.

However, this specific contacts of the head-azaindole moiety with NAMPT could not explain the discrepancy in the considerably weaker inhibitory activity of **3b** than that of **3a** or **1**. Therefore, to better understand this phenomenon, we focused on the importance of linker-motifs for the potencies of NAMPT inhibitors. Thus, we analyzed closely the binding forms of the linker-azacyclohexane motifs of **3a** and **3b** in the tunnel cavity of NAMPT. The carbon atom (C4) of piperidine in **3a** and nitrogen atom (N1) of piperazine in **3b** were revealed to protrude approximately 1Å deeper into the NMA binding active site, as compared to the corresponding carbon atom of piperidine in **1**. Importantly, the electronic repulsion of the replaced nucleophilic N1 atom of piperazine in **3b** with the Nδ atom of the imidazole side chain of His191, located 3.82 Å away from the N1 atom, was revealed to occur (Figure 4c). In contrast, there was no electronic repulsion of the corresponding carbon atom of piperidine in **3a** with the Nδ atom as well as that of **1**. These results clearly show that the electronic repulsion interferes with the proper binding of **3b** to the tunnel cavity of NAMPT. 

It is noteworthy that Zheng and colleagues have reported that the imidazole ring of His191 interacts with the linker aromatic group of GNE series inhibitors through a herringbone stacking [44,45,47]. Furthermore, NAMPT mutations, including H191R, D93del, Q388R, S165Y and G217V, have been reported to confer resistance to NAMPT inhibitors [50,51,52]. In addition, we have recently shown that an NAMPT mutation variant of HCT116 cells generated by continuously exposing cells to increasing concentrations of **1**, which confers cross-resistance to diverse NAMPT inhibitors, such as CHS-828, GNE-617 and STF-118804, was mapped to only H191R [36,37]. Thus, this His191 residue should be considered with higher priority in designing further effective NAMPT inhibitors. That is, it is preferable to avoid interactions with His191 in designing novel NAMPT inhibitors.

### 2.3. Influence of Enthalpic Effects of **3a** and **3b** on the Interactions with Tunnel Cavity of NAMPT

**Figure 5:**
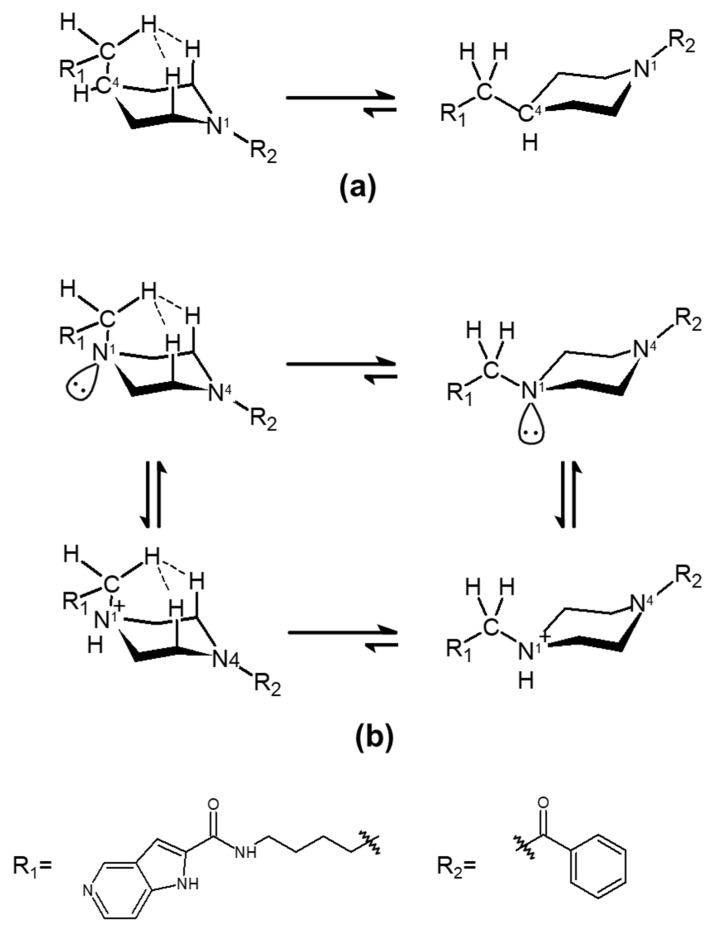
Stable conformers of **3a** and **3b** in the tunnel cavity of NAMPT. The conformational states of **3a** (**a**) and **3b** with its protonation form (**b**) in the tunnel cavity of NAMPT were analyzed by in silico binding mode analysis and crystal structure of **1** (PDB code: 2GVJ) [38,39,40,41,42]. The hydrogen interactions of axial-methylene with 1,3-diaxial hydrogens are represented by dashed lines.

The C-to-N conversion in the azacylohexane-linker moiety was considered to be possible to introduce some entropic differences in the compounds’ abilities to interact with the tunnel cavity of NAMPT. Although piperazine and piperidine rings are known to form various conformations from chairs to half-chair and skewed boat-to-boat forms in aqueous solution [54], piperazine-containing **3b** and piperidine-containing **3a** took nearly identical positions as a chair form to that occupied by **1** in the tunnel cavity analyzed by in silico binding modes and crystal structure analyses [38,39,40,41,42] (Figure 4). In addition, both C4 in **3a** (Figure 5a) and tertiary amine-type nitrogen (N1) containing **3b** (Figure 5b) might prefer equatorial conformations to axial ones by 1,3-diaxial hydrogen interaction [54], taking a stable chair conformation in the tunnel cavity of NAMPT. Therefore, such entropic effects in **3a** and **3b** in the tunnel space of NAMPT may not be so different from each other. Thus, it is possible to consider that the dramatic differences in enzymatic and cellular potencies between **3a** and **3b** may be due to enthalpic effects rather than entropic effects inside the tunnel cavity of NAMPT.

To prove this hypothesis, we attempted to analyze the binding affinity scores of **3a** and **3b** to NAMPT (PDB Code: 2GVJ) by our in silico binding mode analyses using RDKit [48] and LigandScout [49], as described in Materials and Methods. Remarkably, the binding affinity scores of **3a** and **3b** (non-protonation form) (Figure 5b) were calculated to be −29.07 and −22.13 kJ/mol, respectively, while that of **1** (−28.05 kJ/mol) was near to the value of **3b** (Table 1). Thus, the piperidine-motif was revealed to be beneficial for **3a** potency and has valuable influences on the interaction with the tunnel cavity of NAMPT.

The N1 and N4 atoms in **3a** and **3b**, respectively, would take an *sp*^2^-like bond, due to the effect of the proximity of the benzoyl group (R_2_) on the lone pair (Figure 5a,b). In contrast, although it is hard to know whether or not the N1 atom of piperazine in **3b**, which takes an *sp*^3^ bond like the C4 atom in **3a**, would be protonated in the tunnel cavity of NAMPT, it is possible to be protonated in physiological conditions. Thus, the binding affinity score of **3b** in the protonation state was measured by LigandScout. Interestingly, it was calculated to be −23.85 kJ/mol and was not as good as that of the non-protonated form (Table 1). In the protonated N1 atom, the electrostatic interaction becomes attractive to the Nδ of His 191 but repulsive against the protonated Nδ. However, as the imidazole ring of His has aromaticity, the positive charge introduced by protonation does not tend to be localized on the Nδ. Thus, the repulsion may not be so strong. In addition, it seems that a hydrogen bond between the protonated Nδ of His191 and the N1 atom in **3b** could not be formed, because the angle that NδH-N1 (piperazine) takes is 106.4°. Furthermore, of course, the influence of the hydrophobic amino acids in the tunnel region on the protonated N1 in **3b** cannot be ruled out, and they may negatively influence this interaction. Accordingly, the electrostatic enthalpy effects were suggested to interfere with the proper binding of **3b** to the tunnel cavity of NAMPT. For this reason, both the non-protonated and protonated forms of **3b** are suggested to have lower binding affinities to the tunnel cavity of NAMPT than does **3a**. These outcomes indicate that the electrostatic potential (enthalpy term) inside the tunnel region of NAMPT has a significant impact on the different binding affinity of **3b** from that of **3a**.

## 3. Materials and Methods

### 3.1. Enzymatic NAMPT Assay

For the inhibitory effects of **3a** and **3b** on NAMPT activity, recombinant NAMPT activity was measured using a coupled-enzyme reaction system (CycLex NAMPT Colorimetric Assay Kit: MEDICAL & BIOLOGICAL LABORATORIES CO., LTD., Nagoya, Japan) according to the manufacturer’s instructions, as described previously [31,36,37]. Briefly, NAMPT enzyme and tested compounds were put on the 96-well transparent plate. The mixture of the rest of contents (ATP, NAM, PRPP, nicotinamide nucleotide adenylyltransferase 1 (NMNAT1), water-soluble tetrazolium salts (WST-1), alcohol dehydrogenase, diaphorase and ethanol (EtOH)) were then added onto the wells. After a brief incubation, the absorbance of the samples was detected at 450 nm on every 10 min until 60 min using a SYNERGY HTX (BioTek Instruments, Inc.; Winooski, VT, USA). The inhibition rates were calculated by the initial slope of the absorbance of the tested samples divided by that of the control wells. The IC_50_ values for the samples were calculated by 4-parameter logistic regression using Image J 1.48v (https://imagej.nih.gov/ij/).

### 3.2. Cells and Cell Culture

HCT116 cell was obtained from American Type Culture Collection (ATCC) (Manassas, US) and cultured in Dulbecco’s modified Eagle medium (DMEM) (FUJIFILM Wako Pure Chemical Corporation) supplemented with 10% of fetal bovine serum (Biosera Europe; Nuaillé, France), 100 Units/mL of penicillin G and 100 μg/mL of Streptomycin (FUJIFILM Wako Pure Chemical Corporation) [36,37].

### 3.3. Cell Activity Assay

One thousand HCT116 cells were inoculated into the 96-well transparent cell culture plate and cultured for 24 h. The medium was changed to new medium that contained the tested compounds and was further cultivated for 72 h. Following the culture, 1/10 volume of the WST-8 reagent (FUJIFILM Wako Pure Chemical Corporation) was added and incubated for 1 h. After the incubation, the absorbance of the samples was detected at 450 nm using a SYNERGY HTX (BioTek Instruments, Inc.). Cell activity was expressed as percentage of control cells treated with vehicle alone [31,36,37].

### 3.4. In Silico Binding Mode and Binding Affinity Score Analyses

The crystal structure of NAMPT was downloaded from the Protein Data Bank (PDB code: 2GVJ) for molecular binding mode analyses. In silico binding mode analyses were done using RDKit [48] and LigandScout [49]. First, 1000 conformations of each inhibitor were generated using the EmbedMultipleConfs and MMFFGetMoleculeForceField functions of RDKit. Second, the conformations of each inhibitor were aligned to the crystal structure of **1** using Open3DALIGN [53] implemented in RDKit. The conformation of each inhibitor, which has the highest alignment score, was selected. Third, energy minimization of the selected conformation of each inhibitor with NAMPT was performed, and then the binding affinity score of each inhibitor was calculated using LigandScout [49].

### 3.5. Synthetic Procedures of the Target Compounds

#### 3.5.1. General Experimental Methods

^1^H-NMR spectra were recorded at 400 MHz using a JEOL ECZ400 operating (JEOL Ltd.; Tokyo, Japan) at the indicated frequencies. Chemical shifts were expressed in ppm relative to the internal standard tetramethylsilane (ppm = 0.00). All reagents and solvents were purchased from FUJIFILM Wako Pure Chemical Corporation. The progress of all reactions was monitored by TLC using ethyl acetate/hexane, acetone/hexane, dichloromethane (CH_2_Cl_2_)/MeOH and CH_2_Cl_2_/MeOH/triethylamine (TEA) as the solvent system, and spots were visualized by irradiation with ultraviolet light (254 nm) or ninhydrin reaction. Column chromatography was performed using silica gel (200–300 mesh).

#### 3.5.2. General Procedures for Synthesizing the Target Compounds **3a** and **3b**

Compounds 1-benzoylpiperidin-4-yl)butyl)isoindoline-1,3-dione (**1a**) and 2-(4-(4-benzoylpiperazin-1-yl)butyl)isoindoline-1,3-dione (**1b**) were synthesized according to Gilig et al. [55] with slight modifications.

#### 3.5.3. (4-(4-aminobutyl)piperidin-1-yl)(phenyl)methanone (**2a**)

Hydrazine hydrate (0.3 mL, 6.17 mmol) was added under Ar_2_ atmosphere to a solution of **1a** (1.0 g, 2.56 mmol) in EtOH at 20 °C. After stirring at 20 °C for 10 min, the mixture was heated under reflux for 2 h. After cooling to 20 °C, the white precipitate was filtered off and washed with EtOH (10 mL). The EtOH solutions were combined, and the solvent was evaporated in vacuo. The residue was taken in CH_2_Cl_2_ (20 mL) and a saturated aqueous solution of K_2_CO_3_ (20 mL). Vigorous stirring for 10 min produced two clear phases. The aqueous phase was extracted with CH_2_Cl_2_ (10 mL, 3 times). The combined organic phases were washed with brine (30 mL) and dried over Na_2_SO_4_. After solvent evaporation in vacuo, **2a** was obtained (0.530 g) as a pale-yellow oil.

#### 3.5.4. *N*-(4-(1-benzoylpiperidin-4-yl)butyl)-1*H*-pyrrolo[3,2-c]pyridine-2-carboxamide (**3a**)

First, 1*H*-pyrro[3,2-c]pyridine-2-carboxylic acid (0.149 g, 0.918 mmol) was added to a solution of **2a** obtained above (0.239 g, 0.918 mmol) in dry DMF (8.0 mL) in a 25 mL round-bottomed flask. HOBt (0.186 g, 1.38 mmol), EDC-HCl (0.352 g, 1.84 mmol) and N, *N*-diisopropylethylamine (0.462 mL, 2.75 mmol) were added sequentially, and the suspension was allowed to stir at room temperature overnight. The reaction mixture was then poured into a separatory funnel containing water and ethyl acetate. The aqueous phase was separated and extracted three times with EtOAc. The combined organic phase was dried over Na_2_SO_4_, filtered and concentrated in vacuo. The resulting residue was purified by column chromatography (CH_2_Cl_2_/MeOH = 9/1) to afford the target product **3a** (0.215 g, yield 58%, more than 95%, as judged by ODS(C18) HPLC) as white crystals. ^1^H-NMR (DMSO-d6) d 11.94 (s, 1H), 8.88 (s, 1H), 8.59 (t, 1H), 8.19 (d, 1H), 7.39 (m, 3H), 7.31 (m, 3H), 7.20 (s, 1H), 4.43 (m, 1H), 3.51 (m, 1H), 3.26 (m, 2H), 2.96 (m, 1H), 2.70 (m, 1H), 1.70 (m, 2H), 1.51 (m, 3H), 1.29 (m, 4H), 1.05 (m, 2H). 

#### 3.5.5. 4-(4-Aminobutyl)piperazin-1-yl)(phenyl)methanone (**2b**)

Compound **2b** was obtained from **1b** by similar synthetic processes as described in Section 3.5.3.

#### 3.5.6. *N*-(4-(4-Benzoylpiperazin-1-yl)butyl)-1*H*-pyroro[3,2-c]pyridine-2-carboxamide (**3b**)

The procedure was the same as described above for the synthesis of **3a**. Compound **3b** was obtained as pale-yellow powder (0.104 g, yield 28%, more than 95%, as judged by ODS(C18) HPLC). ^1^H-NMR (DMSO-d6) δ 11.97 (s, broad, 1H), 8.90 (s, 1H), 8.61 (t, 1H), 8.19 (d, 1H), 7.33–7.43 (m, 5H), 7.23 (s, 1H), 3.26–3.58 (m, 9H), 2.30–2.38 (m, 5H), 1.48–1.55 (m, 3H).

### 3.6. Statistical Analysis

All data were obtained from at least three independent experiments and were expressed as mean ± standard error (SE).

## 4. Conclusions

In this study, to understand the structural basis of beneficial design for effective NAMPT inhibitors, we designed and synthesized a close structural analogue set of potent and specific azacyclohexane-motif NAMPT inhibitors, namely **3a** (piperidine-motif) and **3b** (piperazine-motif) modified from **1**. In addition, these two compounds have 5-azaindole ring as the head structure, which is replaced by the vinylpyridine ring of **1**. Through biochemical experiments and in silico binding mode analyses using these inhibitors, we clearly showed that **3a** is the most potent NAMPT inhibitor among these inhibitors, and that the azaindole-head and piperidine-linker of NAMPT inhibitors are promising motifs that result in enthalpically higher potent inhibitory activity against NAMPT molecule and cancer cells. The main reason for this phenomenon was revealed to be due to the electronic repulsion of the piperazine-linker motif of **3b** with His191 of NAMPT. Importantly, this H191 is known to be a drug-resistance sensitive amino acid residue [36,37,50,51,52]. Thus, it is better to avoid interactions with His191 in designing effective NAMPT inhibitors. Further studies on the tunnel cavity of NAMPT using rigid analogues of azaindole-piperidine-motif inhibitors will allow a more definitive understanding of the inhibitor pharmacophore. Although additional confirmatory studies, such as the synthesis of novel azaindole-piperidine-motif NAMPT inhibitors and details of in vivo efficacy and resistibility are warranted, our findings provide valuable insights into the design for effective NAMPT inhibitors that offer improved therapeutic potential by making high specificity and avoiding resistance to NAMPT. Furthermore, on the basis of the characteristics of NAMPT functions and the mechanisms of action of NAMPT inhibitors, we are now trying to design additional azaindole–piperidine-motif NAMPT inhibitors available in vivo, such as the translation of a potent one into a payload for antibody–drug conjugates, which are an important therapeutic modality enabling targeted drug delivery to cancer cells [56].

## Figures and Tables

**Table 1:** Biochemical properties and binding affinity scores of **3a** and **3b** NAMPT inhibitors.

Compound	IC_50_^a^ (μM)	Binding Affinity Score ^b^ (kJ/mol)	
		Non-Protonation	Protonation
**3a** (piperidine)	0.11	−29.07	-
**3b** (piperazine)	5.06	−22.13	−23.85
**1** (piperidine)	0.52	−28.05	-

^a^ The IC_50_ values of these three NAMPT inhibitors were determined by the titration curves in Figure 3a. ^b^ The binding affinity scores of these three inhibitors in the tunnel cavity of NAMPT were calculated by LigandScout [49].
